# Functional Reduced-Fat Mozzarella Cheese from “Essential Oil-Fed” Milk and Inulin Fortification

**DOI:** 10.3390/foods15091565

**Published:** 2026-05-01

**Authors:** Claudia Antonino, Giuseppe Natrella, Pietro Caliandro, Lucrezia Forte, Antonella Pasqualone, Michele Faccia

**Affiliations:** 1 Department of Soil, Plant and Foods Science, University of Bari, Via Amendola 165 A, 70126 Bari, Italy; claudia.antonino@uniba.it (C.A.); pietro.caliandro@uniba.it (P.C.); antonella.pasqualone@uniba.it (A.P.); michele.faccia@uniba.it (M.F.); 2 Department of Veterinary Medicine, University of Bari Aldo Moro, Strada Provinciale per Casamassima Km 3, Valenzano, 70010 Bari, Italy; lucrezia.forte@uniba.it

**Keywords:** Mozzarella cheese, inulin, reduced-fat, fat replacer, unsaturated fatty acids

## Abstract

The demand for functional dairy products is increasing, in response to the adverse correlation between high saturated fat consumption and cardiovascular health problems. The present study investigated the physicochemical and sensory features of a prototype of functional reduced-fat Mozzarella cheese fortified with inulin made from milk obtained by integrating the cattle diet with laurel essential oil (LEO). Two samples were compared over a 10-day storage period: a whole-milk Mozzarella cheese (MC), and a reduced-fat Mozzarella cheese fortified with 10% (*w*/*v*) of inulin (MI). The results show that incorporating inulin during the stretching phase required more time (2.55 min longer) to obtain the final product. However, in addition to a 5% fat decrease, the MI cheese achieved an inulin content of 3.31%, satisfying the European Regulation No 1924/2006, for the “Source of Fiber” claim. On the other hand, from a nutritional perspective, the dietary LEO integration significantly modulated the lipid fraction of the sample, resulting in a 40% increase in monounsaturated fatty acids (MUFAs) and a marked enrichment in polyunsaturated fatty acids (PUFAs). Considering the texture attributes, the incorporation of inulin during the stretching phase led to the formation of a micro-gel that acted as a functional filler, resulting in significantly higher hardness (33.41 N for MI and 16.10 N for MC), throughout the 10-day storage period. Temporal Check-All-That-Apply (TCATA) analysis confirmed that while the MI sample introduced vegetable and cooked milk notes, MI maintained major textural integrity throughout the shelf-life. These findings demonstrate that the synergy between inulin fortification and dietary laurel essential oil supplementation represents a highly effective strategy for producing reduced-fat *pasta filata* cheeses. This dual approach not only preserves sensory and textural integrity but also yields a high-value functional product characterized by an optimized fatty acid profile and a significant fiber intake.

## 1. Introduction

In recent years, the dairy industry has faced increasing pressure to develop healthier product alternatives, as consumers are becoming more health-conscious and limiting their intake of saturated fats to improve overall physical well-being [1,2]. Thus, the development of reduced-fat functional foods, particularly through fiber enrichment, represents a strategic industry response to shifting consumer preferences toward health-promoting diets [3]. Among traditional dairy products, Mozzarella cheese is one of the most widely consumed worldwide. However, its traditional formulation is characterized by a significant lipid content, containing more than 60% saturated fatty acids (SFAs) [4]. Developing low-fat or reduced-fat variants of Mozzarella presents substantial technological challenges, as milk fat plays a crucial role in defining the characteristic texture, melting properties, and flavor profile of cheeses [5,6]. The reduction in fat in cheese matrices typically leads to a more compact and rigid para-casein network. In the absence of fat globules, which act as structural disrupters and lubricants, the protein matrix becomes overly dense, resulting in a rubbery texture and poor meltability [7].

Fat replacers are strategically employed to mitigate the rheological and sensory deficiencies typical of reduced-fat formulations. Beyond lowering the caloric density of the food, these constituents enhance the functional and textural attributes of the dairy matrix, effectively simulating the mouthfeel and structural contribution of milk fat [8]. The incorporation of dietary fibers has emerged as a successful strategy. Among these, polysaccharide and fiber-based fat mimetics, such as inulin, play a fundamental role in modulating the textural and functional attributes of dairy products [9]. Inulin exerts a multifunctional role, acting as a low-calorie sweetener, fat substitute, and texturizing agent. Beyond its prebiotic properties, inulin effectively replicates the mouthfeel and sensory profile of fat [10], significantly enhancing the structural integrity of reduced-fat cheeses [11,12,13,14]. These functional properties are strictly dependent on the degree of polymerization (DP). Specifically, long-chain inulin exhibits lower solubility and higher viscosity compared to its native counterpart, acting as a potent texture modifier. The DP also regulates critical physicochemical parameters, such as the melting transition temperature, gel-forming capacity, and overall gel strength, as well as the synergy with other food constituents like water and protein [13]. Given that Mozzarella is a cheese produced through a high-temperature stretching process [15], inulin solubilization mimics the mouthfeel of fat and provides structural resilience [5,16]. Recent studies on various cheese matrices suggest that beyond its structural role [6,11,17,18,19], inulin also acts as a prebiotic, fostering the growth of beneficial lactic acid bacteria and thus enhancing the overall functional value of the product [20,21,22].

Beyond technological fortification, the nutritional profile of dairy products can be further enhanced through upstream interventions in animal nutrition. The use of vegetable extracts and essential oils in dairy cattle diets has been recognized as an innovative tool to modulate ruminal fermentation and improve the fatty acid composition of milk [23]. Specifically, the supplementation of essential oils, such as laurel oil, rich in eugenol and terpenes, can influence the biohydrogenation of unsaturated fatty acids in the rumen, leading to an enrichment of the milk fat with health-promoting compounds, including monounsaturated and polyunsaturated fatty acids [24,25]. A higher proportion of health-promoting MUFAs and PUFAs, such as oleic and linoleic acids, is absorbed and subsequently secreted into the milk [26].

The application of inulin as a fat replacer in Mozzarella cheese has been investigated by Ahmad et al. [27], who observed that its inclusion in low-fat Mozzarella made from buffalo milk effectively reduced the meltability and stretchability typically associated with low-fat formulations, enhancing both hardness and chewiness. Similarly, Moghiseh et al. [11] reported that inulin acts as a structural modifier by increasing moisture retention and protein content within the cheese matrix; their findings highlighted that while high concentrations of inulin significantly increase hardness, they also lead to lower springiness and higher cohesiveness, which are crucial for achieving a desirable mouthfeel in low-fat variants. Furthermore, Li et al. [28] demonstrated that inulin serves as an effective plasticizer in reduced-fat Mozzarella-like products, decreasing hydrophobic interactions within the casein matrix and contributing to a more flexible protein network. While many studies have investigated fat replacers or dietary supplements independently, comprehensive research on the effects of combining animal dietary interventions with plant-based fiber fortification is still lacking. This approach aims to create a product that is not only lower in calories but also improved in its lipid profile and structural integrity.

The aim of this study was to evaluate the combined impact of dietary laurel essential oil supplementation in dairy cows and the addition of long-chain inulin on the physicochemical, textural, and sensory properties of reduced-fat Mozzarella cheese. By analyzing the chemical and structural evolution and sensory profile over a 10-day storage period, this research aimed to produce functional *pasta filata* cheese that meets nutritional requirements while maintaining high sensory quality.

## 2. Materials and Methods

### 2.1. Cheese Manufacturing

Cheesemaking trials were conducted at a local dairy farm located in Ceglie Messapica (Masseria Fragnite, Apulia, Southern Italy). Three independent replicates were prepared for each sample type of Mozzarella cheese, following the method described by Antonino et al. [29], with slight modifications.

The control Mozzarella sample (MC) was produced from whole cow’s milk (3.90% fat; 3.65% protein), whereas milk for the experimental samples (MI) derived from cows orally supplemented with 3 mL of pure LEO per day for two weeks. The LEO used in the feeding trial was supplied by Bioplanta (Irsina, Matera, Italy). The same batch used for cow supplementation was chemically characterized by gas chromatography–mass spectrometry (GC–MS) using an HP 6890 gas chromatograph coupled to an HP 5972 mass selective detector and equipped with an HP-5MS capillary column (30 m × 0.25 mm i.d.; 0.25 μm film thickness). Helium was used as the carrier gas. The injector temperature was set at 250 °C, with a split ratio of 50:1. The oven temperature program was as follows: from 60 to 110 °C at 2 °C/min, followed by an increase from 110 to 220 °C at 10 °C/min. Individual compounds were identified by comparing their mass spectra with data from the literature and by matching their gas chromatographic retention indices with those reported by Adams [30]. The relative abundance of each compound was expressed as a percentage of the total chromatographic area. GC–MS analysis identified 84.25% of the total chromatographic composition. The oil showed a chemically complex profile dominated by oxygenated monoterpenes, followed by monoterpene hydrocarbons, phenylpropanoids, and minor sesquiterpenes. The main identified constituents were 1,8-cineole (16.87%), linalool (14.71%), α-terpinene (10.73%), cis-sabinene (7.23%), eugenol (7.07%), α-pinene (6.41%), methyleugenol (3.00%), and terpinen-4-ol (2.47%). Other minor compounds accounted for the remaining identified fraction, whereas 15.75% of the chromatographic area remained unidentified.

Prior to cheesemaking, the raw milk was partially skimmed (2.65% fat; 3.56% protein) using a stainless-steel vertical centrifugal separator (Pieralisi Maip S.P.A., Jesi, Italy), operating at 5000 rpm for 5 min. The process was conducted in continuous mode with an inlet temperature of about 30 °C. Milk composition was monitored before and after the skimming process using MilkoScan analysis (FoodScan™ 2 Lab/Pro, FOSS Analytics A/S, Hillerød, Denmark) to ensure the target final fat content.

The cheesemaking process was similar for both samples except for inulin inclusion during the stretching phase for MI Mozzarella. In brief, raw cow’s milk was cold-acidified to pH 5.85 using citric acid, then heated to 36 °C via direct steam injection. Subsequently, 20 mL/100 L of the coagulant Chymostar^®^ (Danisco Italy S.P.A., Trezzano sul Naviglio, Italy) was added, resulting in coagulation within approximately 25 min. The curd was manually cut using a curd breaker (spino), with the temperature raised to approximately 38 °C. After cutting, the curd was allowed to rest for 30 ± 10 min before transferring to draining tables for whey expulsion. For all samples, the curd obtained (10 kg for each formulation) was finely chopped, salted at 40 g/kg, and manually stretched with 10 L of water at 95 °C to form ~100 g Mozzarella spheres. The stretching phase was performed at pH 5.80. For the MI sample, long-chain inulin (Gioia Group S.r.l., Torino, Italy) was dissolved in the stretching water at 10% (*w*/*v*), followed by stretching under the same conditions. All samples were individually packaged in plastic pots, fully immersed in water, sealed with plastic film, and stored at 4 ± 2 °C.

### 2.2. Stretching Parameters

During cheesemaking, the following parameters were monitored: (i) stretching time (min), defined as the interval from hot water addition to curd until Mozzarella molding initiation; (ii) residual stretching water (L), the volume remaining after complete stretching and molding; and (iii) Mozzarella yield (kg), the mass obtained post-stretching.

### 2.3. Cheese Characterization

#### 2.3.1. Chemical Composition

Proximate composition was determined using a benchtop FoodScan™ NIR spectrophotometer (850–1100 nm; FoodScan™ 2 Lab/Pro, FOSS Analytics A/S, Hillerød, Denmark) with commercial calibration models. Fat, protein and fiber contents were confirmed using AOAC methods 979.09, 945.38, and, 991.43, respectively [31].

The pH was measured with a pH meter (HANNA Instruments, Woonsocket, RI, USA). Moisture content was monitored during 10 days of storage according to AOAC method 950.46.

Lactose was quantified following Natrella et al. [32]. Briefly, 10 g of minced Mozzarella was mixed with 20 mL Milli-Q water, stirred for 1 h, centrifuged at 6000 rpm for 10 min at 4 °C, and filtered (0.2 μm syringe filter). Aliquots (10 μL) were analyzed by HPLC-RID (Agilent Technologies, Palo Alto, CA, USA) on a Rezex™ RCM-Monosaccharide column (300 × 7.8 mm, Phenomenex, Torrance, CA, USA) held at 80 °C. Isocratic elution used Milli-Q water (Millipore Corp., Bedford, MA, USA) at 1 mL/min. Quantification employed external calibration (0.01–50 mg/mL) with pure analytical standard (Sigma-Aldrich, Milan, Italy).

The lipid fraction was extracted according to Antonino et al. [33] using a Soxhlet apparatus (SER 148 extraction system, Velp Scientifica S.r.l., Usmate, Italy) with ethyl ether as the solvent (Carlo Erba, Milan, Italy). Fatty acid composition was determined as described by Squeo et al. [34] on a 7890A gas chromatograph (Agilent Technologies, Santa Clara, CA, USA) equipped with an FID detector (220 °C) and an SP™-2340 capillary column (60 m × 0.25 mm i.d., 0.20 μm film thickness; Supelco, Bellefonte, PA, USA). Fatty acids were identified by retention time comparison to a C4-C24 standard mixture (Sigma-Aldrich, St. Louis, MO, USA), and expressed as a percentage.

#### 2.3.2. Texture and Color Evolution

Texture was assessed using a Z1.0 TN texture analyzer (Zwick Roell, Ulm, Germany) equipped with a 4 cm stainless-steel square probe and a 50 N load cell. Data were acquired via TestXpert^®^ II version 3.41 software (Zwick Roell). Mozzarella cubes (2 cm^3^) underwent double-compression texture profile analysis (TPA) with the following parameters: 1 mm/s compression speed, 50% deformation in both cycles, and a 5 s delay before the second compression [29]. The TPA parameters included: (i) hardness (N; peak force of first compression); (ii) springiness (the ratio of the time difference during the second compression to that during the first compression); (iii) cohesiveness (ratio of positive force area in second to first compression); and (iv) chewiness (N; hardness × springiness × cohesiveness).

Color was evaluated using a CM-600d colorimeter (Konica Minolta, Tokyo, Japan) and SpectraMagic NX2 software, as described by Antonino et al. [35]. Brightness (L*), red index (a*), and yellow index (b*) were considered in accordance with the International Commission on Illumination (CIE).

#### 2.3.3. Sensory Evaluation

Sensory analyses were performed by a trained panel of five experts from the Italian Association of Cheese Tasters (ONAF), each with ≥5 years of experience and selected following ISO 8586:2012 (International Organization for Standardization, 2016) [36]. Quantitative descriptive analysis was conducted, with assessors scoring samples for specific odor and taste attributes on a 5-point scale (0–4), in accordance with Antonino et al. [37]. Evaluated attributes comprised: two appearance descriptors (color and texture uniformity); four orthonasal/retro-nasal sensations (fresh milk, cooked milk, sour milk, and vegetable); four taste flavor attributes (sweetness, saltiness, acidity, and sourness); and five texture descriptors (firmness, adhesiveness, springiness, solubility, and greasiness). Evaluations were performed under blind conditions in a sensory laboratory equipped with individual cabins.

### 2.4. Statistical Analysis

Minitab19 (Minitab Inc., State College, PA, USA) was used for the statistical analysis of all results, reported as mean ± standard deviation (SD) of three replications. To evaluate the differences between samples, one-way and two-way ANOVA followed by Tukey’s HSD test for multiple comparisons was applied. Error bars with fill area and 3D waterfall plot were performed using OriginPro (OriginLab, Northampton, MA, USA). Finally, Temporal Check-All-That-Apply analysis (TCATA) for sensory analysis was performed using Xlstat (Addinsoft, France).

## 3. Results and Discussion

### 3.1. Impact of Reduced Fat and Fiber Addition on Stretching Parameters

The evaluation of the stretching parameters (Table 1) indicated that both fat reduction and the incorporation of long-chain inulin significantly influenced the processing time required for the final product. Specifically, stretching time was the only parameter found to be significantly higher (*p* < 0.05) in the MI samples, while residual stretching water and Mozzarella yield remained unchanged. In the MC, fat globules were interspersed within the casein network; during the stretching process, the fat acted as a natural lubricant, facilitating the sliding of protein fibers over one another with minimal resistance [38]. Conversely, in MI samples, the combination of reduced-fat content and increased viscosity of the stretching water—attributed to the presence of inulin—hindered the alignment of protein fibers. This resulted in a harder and more springy curd texture [39,40]. These findings are consistent with the existing literature [38,39], which suggests that extended stretching times are necessary to achieve acceptable functional properties in reduced-fat cheese products. Overall, the stretching behavior of the Mozzarella formulated with inulin as a fat replacer was governed by the viscosity of the inulin and the absence of the lubricating fat phase.

### 3.2. Physicochemical Characteristics of Mozzarella Cheese

As expected, the gross composition of freshly produced Mozzarella (Table 2) changed (*p* < 0.05) upon the addition of long-chain inulin and the reduction in milk fat. Indeed, the addition of 10% (*w*/*v*) inulin during the stretching phase resulted in about one/third retention (3.31%) in MI cheese, fulfilling the criteria for the “Source of Fiber” claim (Regulation (EC) No 1924/2006) and confirming the effectiveness of the technological incorporation process reported in previous studies [29], even when using semi-skimmed milk. This result was similar to that reported in the literature by Juan et al. [41], who used 5% (*w*/*v*) of inulin to produce reduced-fat fresh cheese, achieving a final retention of 3.01%. Furthermore, moisture was not significantly different among the samples, contrary to Moghiseh et al. [11], who used 6% *w*/*w* inulin solution added in milk for Mozzarella production and hot water at 70–80 °C for the stretching phase, and attributed the higher moisture observed to the greater water absorption of the added polysaccharide. The fat content in the MI sample was 22.27% lower, whereas the protein content was 9.81% higher than the MC sample [41].

**Figure 1:**
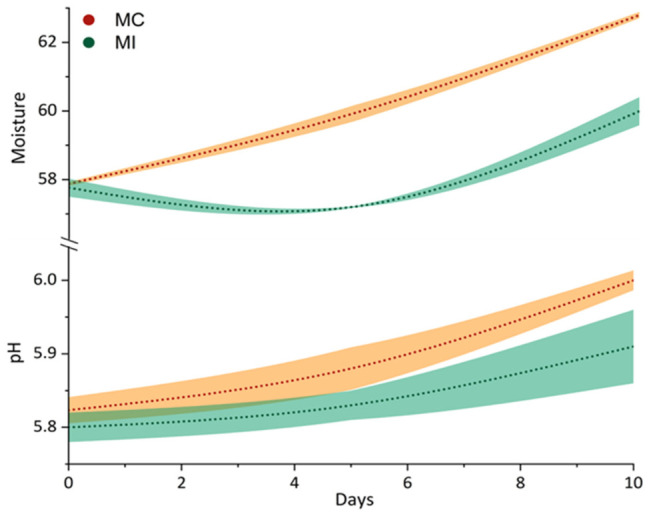
Error bars with SD fill area of Mozzarella pH value and moisture (%) during 10 days of storage. Abbreviations: MC, whole-milk Mozzarella cheese control; MI, reduced-fat Mozzarella cheese with inulin.

The results of the pH monitoring (Figure 1) showed greater similarity between MC and MI at day 0 of the storage period. Indeed, in the experimental sample, the pH increased after production, in line with Islam et al. [42]; however, this parameter increased in both samples up to 10 days of storage. This observation may be attributed to the matrix’s sustained water absorption capacity, which induced a dilution effect promoted by proteolytic activity. This, in turn, facilitated the release of low-molecular-weight compounds with basic properties [43]. These data are confirmed by the increase in moisture over time for both samples, in accordance with Antonino et al. [29].

Variations in lactose concentrations served as chemical indicators of microbial metabolic activity [44]. During the early stages of cheesemaking, starter cultures facilitate the bioconversion of lactose into galactose and glucose, which are subsequently metabolized into lactic acid and various secondary metabolites [45]. Our findings suggest that inulin addition and reduced milk fat influenced the lactose profile of the samples (*p* < 0.05). Specifically, the inulin-fortified samples (MI) exhibited higher residual lactose percentage levels (0.95 ± 0.03) compared to the control (0.40 ± 0.03). Both samples exhibited a reduction in lactose content up to the tenth day of storage, reaching values of 0.26 ± 0.06 for MC and 0.75 ± 0.04 for MI. The inulin solution, characterized by high viscosity [46], may have hindered the efficiency of the whey drainage process during stretching; this effect, combined with the fiber’s capacity to bind the aqueous phase [9], promoted the entrapment of whey and its primary solutes—most notably lactose—within the protein matrix [5,16]. Furthermore, the reduction in fat in the proximate composition led to a proportional increase in the concentration of the remaining constituents, including lactose. The fatty acid profile of the Mozzarella samples (Table 3) revealed significant variations attributable to both the dietary supplementation of the cows with LEO and the partial skimming of the experimental milk. The control sample (MC), derived from whole milk with 3.90% fat, exhibited a significantly higher total saturated fatty acid content of 70.14%, whereas the inulin-fortified, reduced-fat sample (MI) showed a reduced SFA concentration of 46.60%. This shift is primarily driven by the marked decrease in individual saturated chains such as palmitic (C16:0), stearic (C18:0), and myristic (C14:0) acids in the MI. Conversely, the MI sample demonstrated a substantial enrichment in monounsaturated fatty acids, which reached 43.70% compared to 25.74% in MC, largely due to a nearly twofold increase in oleic acid (C18:1). Furthermore, the PUFA fraction was significantly higher in MI (9.70%) than in MC (4.12%), specifically regarding linoleic acid (C18:2). It should be noted that while the skimming process reduced the overall fat content, the marked variation in fatty acid distribution in MI is not a direct result of mechanical skimming, but rather a reflection of the modified milk composition induced by the LEO feed. Indeed, the oral administration of laurel essential oil to the livestock exerted a modulating effect on the milk fat synthesis or ruminal biohydrogenation processes [23]. Essential oils are known to alter microbial activity in the rumen, potentially favoring the accumulation of unsaturated intermediates that are subsequently incorporated into the milk fat [47]. This dietary influence, combined with the reduction in the total fat content to 2.65% in the experimental milk, resulted in a more favorable nutritional profile for the MI cheese. From a structural perspective, the decrease in SFAs and the concomitant rise in unsaturated chains in MI might also correlate with the textural observations. While the inulin provides a firming “filler” effect, the modified lipid fraction is characterized by a lower melting point, which potentially influences plasticization during the stretching phase at 85 °C. Consequently, the MI Mozzarella achieves a unique balance between the structural reinforcement provided by the prebiotic fiber and a lipid profile that is significantly improved from a nutritional perspective.

**Figure 2:**
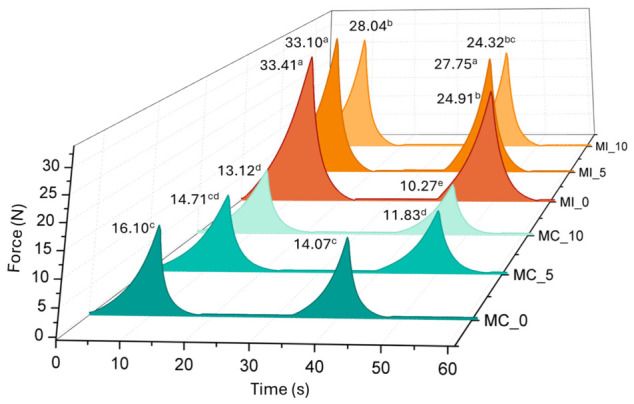
Three-dimensional waterfall plot for hardness (N) of whole-milk control Mozzarella cheese (MC) and reduced-fat Mozzarella cheese with inulin (MI) during storage. ^a–e^ Values for different time/type of Mozzarella cheese bearing different letters are different at *p* < 0.05. Values shown are mean.

The textural properties of the Mozzarella samples were significantly (*p* < 0.05) influenced by the incorporation of inulin and the reduction in lipid content. As illustrated in the 3D waterfall plot (Figure 2), the hardness of MI was higher than that of the control (MC) at all time points. At day 0, MI exhibited a peak force of 33.41 N, more than double the value recorded for MC (16.10 N), in agreement with Antonino et al. [29]. This increase in hardness can be attributed to the “filler effect” of the fiber within the protein matrix, as mentioned above. While milk-fat globules typically act as disrupters of the casein network, providing softness and lubricity [48], their reduction—combined with the introduction of inulin—leads to a more compact and rigid structure, in accordance with Ahmad et al. [27] and Moghiseh et al. [11]. The inulin likely forms a secondary network or fills the interstitial spaces between casein micelles, increasing the resistance to compression, which other studies had also observed [18,29,49]. Throughout the 10-day storage period, both samples showed a progressive decrease in hardness (*p* < 0.05), likely due to primary proteolysis and the solubilization of calcium from the casein micelles into the governing liquid, which weakens the cross-links within the casein network [50]. However, the MI samples retained a significantly firmer structure at day 10 compared to the MC samples at day 0. This structural preservation effect suggests that the inulin–water–protein interaction is less susceptible to the typical softening induced by governing liquids than the traditional fat–casein matrix. The structure of cheese is of pivotal importance when considering its culinary applications (e.g., low-moisture Mozzarella). In this context, meltability represents a key functional property. This characteristic is closely related to the behavior of the protein matrix during heating: as the entrapped fat melts, protein–protein interactions weaken, allowing molecular mobility within the matrix and, consequently, cheese flow [51]. Accordingly, meltability is inversely related to the hardness of Mozzarella. According to the literature, fat reduction and the addition of inulin as a fat replacer lead to decreased meltability in low-moisture Mozzarella [11,28], making it more suitable as a ready-to-eat product intended for direct consumption, as it better maintains its structural integrity during storage. In contrast, for high-moisture Mozzarella, our results are promising. Since this type of cheese is typically consumed fresh, preserving its structure for a longer time represents a desirable quality attribute.

The second compression peak in MI samples is particularly revealing. In the texture profile analysis, the ratio between the second and first peak is a measure of cohesiveness. The fact that MI maintains a high second peak suggests that the inulin–protein complex creates a resilient network. Unlike milk fat, which acts as a lubricant and promotes structural fracture [51], the long-chain inulin molecules likely interpenetrate the casein network, acting as a structural reinforcement that remains intact even after the first deformation. Furthermore, the high stretching temperature (85 °C) facilitates the formation of an inulin micro-gel [9]. This gel fills the serum pockets, exerting a hydrostatic pressure that opposes the compression, thereby increasing the recorded force values. According to the second peak, MC showed a reduction in cohesiveness (Table 4) values during storage (from 0.71 to 0.61), while the inulin-fortified samples exhibited a more stable trend, even increasing (*p* < 0.05) by day 10 (0.70). The impact of inulin and reduced milk fat on springiness was limited, although MI initially showed slightly higher values (0.86), according to Moghiseh et al.’s [11] results. The lack of significant variation over time suggests that the elastic recovery of the Mozzarella is more dependent on the primary protein scaffold than on the added fiber or storage period. Finally, chewiness, which is defined as the energy required for chewing, decreased during storage in both samples (*p* < 0.05), with higher value for MI, according to the firmness value. Despite significant structural shifts in the MI, the specific roles of fat reduction and inulin addition practices, as well as their combined synergistic impact, cannot be separately quantified.

The results of the colorimetric analysis are summarized in Table 5. MC exhibited the highest L* values, whereas lightness significantly decreased following the incorporation of inulin and the reduction in milk fat. In dairy matrices, light scattering by milk-fat globules is primarily responsible for the characteristic white appearance [52,53]. Although particulate inulin can function as a light-scattering agent to enhance opacity [41], its specific impact in this study was influenced by the processing conditions. Specifically, the L* values increased over the 10-day storage period, consistent with the findings of Antonino et al. [29]. In contrast, full-fat cheese control (MC) displayed higher redness (a*) and lower yellowness (b*) indices compared to reduced-fat cheese (MI). This shift may be attributed to the solubilization of inulin in water at 85 °C during the stretching phase; the interaction with curd proteins likely facilitated greater hydration of the fiber, altering the chromatic properties of the original white powder [16,54]. Such hydrothermal treatment suggests that inulin, when fully integrated into the protein matrix rather than remaining in its native particulate form, exerts a distinct influence on the final color profile of the Mozzarella. This mechanism potentially explains the variations in a* and b* indices observed across the fortified samples. Furthermore, a reduction in both a* and b* parameters was noted during storage. Likewise, Sousa et al. [55] and Sedaghati et al. [56] highlighted that a* and b* values decreased over the storage time for the kefir and ricotta cheese. This trend reflects complex interactions between the cheese constituents and the fibers, the influence of the governing liquid, and progressive physicochemical degradation, all of which contribute to chromatic shifts along the yellow-blue (positive b*) and red-green (negative a*) axes [57,58].

**Figure 3:**
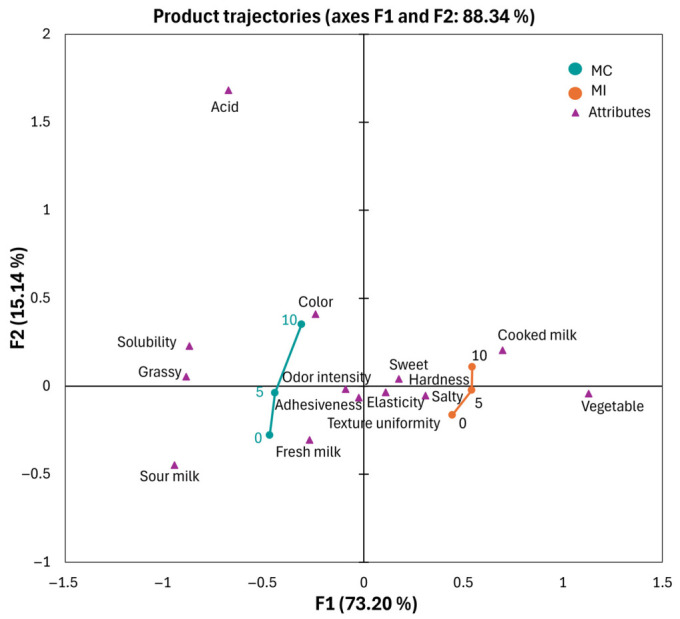
Sensory evaluation by Temporal Check-All-That-Apply of Mozzarella cheese during storage. MC, whole-milk Mozzarella cheese control; MI, reduced-fat Mozzarella cheese with inulin.

The sensory evolution of the Mozzarella samples, analyzed through Temporal Check-All-That-Apply (TCATA, Figure 3) and visualized via a PCA biplot, highlights a clear discrimination between the two experimental groups, which accounted for over 88% of the total variance, with PC1 explaining the highest percentage (73.20%). The MC remained situated within the quadrants associated with fresh milk, sour milk, and grassy notes, shifting toward increased acid and solubility descriptors by the end of the 10-day storage, crossing from negative PC1 to the positive side of PC2. In contrast, the MI sample occupied a distinct sensory space characterized by vegetable and cooked milk attributes. This vegetable note is a direct sensory reflection of the inulin characteristic, while the sweet descriptor aligns with the powerful sweetener of inulin and higher residual lactose levels previously identified in the chemical analysis. Furthermore, the absence of lipid precursors, the lack of fats acting as solvents for characteristic volatile aromatic compounds, and the inhibition of enzymatic reactions essential for the formation of flavor molecules contributed to the distinct aromatic profile of the MI samples. The MI remained situated within the positive side of PC1, with increased cooked milk and vegetable descriptors by the end of the 10-day storage. It is useful to highlight that these two descriptors that emerged in the MI samples did not make the sample unacceptable since they are not unpleasant odors. The proximity of the MI samples to the hardness and elasticity vectors provides sensory validation for the instrumental texture analysis, confirming that the inulin micro-gel successfully compensates for the lack of fat by providing a firm and cohesive mouthfeel. Furthermore, the stability of the MI trajectory relative to texture uniformity suggests that the high-temperature stretching process effectively integrated the fiber into the protein matrix, mitigating the structural softening typically induced by the governing liquid during shelf-life. As storage progressed to day 10, both trajectories moved upward on the F2 axis, although they remained separate. This shift reflects the progressive physicochemical degradation and interaction with the governing liquid, leading to increased intensity in odor and shifts in chromatic perception. However, the MI samples maintained a more stable positioning relative to textural attributes, suggesting that the inulin–protein complex provides a degree of structural resilience against the softening typically induced by storage. This structural characteristic enhances the product’s stability over time. Furthermore, this MI feature did not prove to be a limiting factor for product acceptability; rather, it allowed for the classification of the product as a cheese more suitable for direct consumption and for food processing applications that require lower stretchability and higher structural resistance. It should also be noted that in the field of fresh *pasta filata* cheeses, textural variability is high, as it depends on production technology, raw materials, and the specific manufacturer, other than consumers’ habits [59]. This variability results in a wide diversification of products on the market, allowing consumers to choose according to their individual preferences. In conclusion, the TCATA analysis confirms that while the MI samples diverge from the traditional sensory profile of whole-milk Mozzarella—introducing specific vegetable and cooked milk notes—they successfully overcome the textural deficiencies of reduced-fat cheese by maintaining superior hardness and uniformity throughout the refrigerated storage.

## 4. Conclusions

The current study shows that combining downstream technology fortification with milk fat modification by cattle diet is a valuable approach to producing healthier Mozzarella cheese. From a technological point of view, the incorporation of long-chain inulin during the stretching phase at 85 °C successfully mitigated the typical structural defects associated with fat reduction. The formation of a hydrothermal inulin micro-gel provided a resilient secondary network that filled the serum pockets, imitating the lubricating properties of fat globules and preventing the excessive gumminess often found in reduced-fat *pasta filata* cheeses. Notably, the MI samples maintained superior textural stability and hardness throughout the 10-day storage period, while containing 3.31% of fiber, satisfying the “Source of Fiber” nutritional claim according to Regulation (EC) No 1924/2006. Regarding the sensory characteristics, despite the introduction of subtle vegetable and cooked milk aromatic notes, the cheese preserved a balanced sensory profile and high textural uniformity. In conclusion, the dual approach of integrating essential oil-based cattle diets with inulin fortification bridges the gap between the demand for healthier, low-calorie dairy options and the necessity of maintaining traditional sensory and textural standards. This innovation provides a robust model for the dairy industry to produce functional foods that meet modern dietary guidelines without compromising on quality or consumer satisfaction. Despite the promising results, it must be acknowledged that the current experimental design does not allow for clear discrimination between the individual contributions of fat reduction, LEO supplementation, and inulin addition. Consequently, the potential synergistic effects cannot be rigorously verified at this stage. Future research, employing a multi-factorial design, is required to isolate the impact of each single variable and to characterize their actual interactions in shaping the final product properties. However, it may be necessary to evaluate the melting properties and study the volatile profile variations when constituents of Mozzarella are modified.

## Figures and Tables

**Table 1:** Stretching parameters of Mozzarella cheese during cheesemaking.

Sample	Stretching Time (min)	Residual Stretching Water (L)	Mozzarella Yield (kg)
MC	6.55 ± 1.01 ^b^	9.97 ± 0.31 ^a^	10.10 ± 0.07 ^a^
MI	9.10 ± 0.29 ^a^	9.90 ± 0.10 ^a^	10.18 ± 0.23 ^a^

^a,b^ Values in the same column bearing different letters are different at *p* < 0.05. The values shown are mean ± SD. Abbreviations: MC, whole-milk Mozzarella cheese control; MI, reduced-fat Mozzarella cheese with inulin.

**Table 2:** Proximate composition (expressed as percentage) of fresh Mozzarella cheese.

Sample	Moisture	Fat	Protein	Fiber	Ash
MC	57.88 ± 0.09 ^a^	20.34 ± 0.12 ^a^	19.35 ± 0.13 ^b^	0.02 ± 0.01 ^b^	2.33 ± 0.13 ^a^
MI	57.76 ± 0.11 ^a^	15.81 ± 0.06 ^b^	21.15 ± 0.11 ^a^	3.31 ± 0.05 ^a^	2.27 ± 0.08 ^a^

^a,b^ Values in the same column bearing different letters are different at *p* < 0.05. The values shown are mean ± SD. Abbreviations: MC, whole-milk Mozzarella cheese control; MI, reduced-fat Mozzarella cheese with inulin.

**Table 3:** Fatty acid profile (expressed as percentage) of Mozzarella cheeses.

Sample	MC	MI
C4:0	0.85 ± 0.03 ^a^	0.70 ± 0.02 ^b^
C6:0	1.32 ± 0.01 ^a^	0.78 ± 0.01 ^b^
C8:0	1.11 ± 0.01 ^a^	0.65 ± 0.03 ^b^
C10:0	2.95 ± 0.02 ^a^	1.79 ± 0.01 ^b^
C12:0	3.67 ± 0.01 ^a^	2.28 ± 0.02 ^b^
C13:0	0.14 ± 0.01 ^a^	0.10 ± 0.01 ^b^
C14:0	11.96 ± 0.01 ^a^	7.05 ± 0.01 ^b^
C14:1	1.11 ± 0.02 ^a^	0.69 ± 0.01 ^b^
C15:0	1.28 ± 0.02 ^a^	0.83 ± 0.02 ^b^
C16:0	34.58 ± 0.06 ^a^	23.34 ± 0.04 ^b^
C16:1	0.56 ± 0.01 ^a^	0.06 ± 0.01 ^b^
C17:0	0.62 ± 0.02 ^a^	0.39 ± 0.01 ^b^
C18:0	11.50 ± 0.11 ^a^	8.42 ± 0.01 ^b^
C18:1	23.86 ± 0.04 ^b^	42.83 ± 0.03 ^a^
C18:2T	0.29 ± 0.02 ^a^	0.16 ± 0.01 ^b^
C18:2	3.17 ± 0.01 ^b^	9.05 ± 0.04 ^a^
C20:0	0.15 ± 0.02 ^b^	0.26 ± 0.01 ^a^
C18:3	0.47 ± 0.02 ^a^	0.25 ± 0.01 ^b^
C21:0	0.02 ± 0.01 ^a^	0.02 ± 0.02 ^a^
C20:2	0.04 ± 0.01 ^a^	0.02 ± 0.02 ^b^
C20:3	0.14 ± 0.01 ^a^	0.15 ± 0.01 ^a^
C20:3	0.01 ± 0.04 ^b^	0.06 ± 0.01 ^a^
C22:1	0.20 ± 0.01 ^a^	0.12 ± 0.01 ^b^
∑SFA	70.14 ± 0.02 ^a^	46.60 ± 0.04 ^b^
∑MUFA	25.74 ± 0.06 ^b^	43.70 ± 0.07 ^a^
∑PUFA	4.12 ± 0.01 ^b^	9.70 ± 0.02 ^a^

^a,b^ Values in the same line bearing different letters are different at *p* < 0.05. The values shown are mean ± SD. Abbreviations: MC, whole-milk Mozzarella cheese control; MI, reduced-fat Mozzarella cheese with inulin; ∑SFA, sum of saturated fatty acids; ∑MUFA, sum of monounsaturated fatty acids; ∑PUFA, sum of polyunsaturated fatty acids.

**Table 4:** Texture parameters of Mozzarella cheese during storage.

Sample	Days	Springiness	Chewiness (N)	Cohesiveness
MC	0	0.84 ± 0.01 ^a^	9.22 ± 1.74 ^b^	0.71 ± 0.01 ^a^
MC	5	0.81 ± 0.03 ^a^	8.43 ± 2.13 ^b^	0.68 ± 0.04 ^b^
MC	10	0.83 ± 0.02 ^a^	4.41 ± 2.41 ^c^	0.61 ± 0.04 ^c^
MI	0	0.86 ± 0.01 ^a^	21.20 ± 3.55 ^a^	0.67 ± 0.06 ^b^
MI	5	0.86 ± 0.04 ^a^	15.97 ± 1.99 ^ab^	0.68 ± 0.04 ^b^
MI	10	0.83 ± 0.01 ^a^	16.01 ± 1.03 ^ab^	0.70 ± 0.03 ^a^
S		ns	**	*
T		ns	*	*
S*T		ns	**	*

^a–c^ Values in the same column bearing different letters are different at *p* < 0.05. The values shown are mean ± SD. Abbreviations: MC, whole-milk Mozzarella cheese control; MI, reduced-fat Mozzarella cheese with inulin; ns, not significant; S, type of sample; T, time of storage; *, *p*-value < 0.05; **, *p*-value < 0.001.

**Table 5:** Color parameters of Mozzarella cheese during storage.

Sample	Days	L*	a*	b*
MC	0	90.94 ± 0.62 ^ab^	−0.80 ± 0.04 ^a^	11.84 ± 0.27 ^b^
MC	5	91.43 ± 0.04 ^a^	−0.68 ± 0.06 ^b^	11.43 ± 0.29 ^c^
MC	10	91.20 ± 0.98 ^a^	−0.66 ± 0.08 ^bc^	11.49 ± 0.83 ^c^
MI	0	89.21 ± 0.23 ^c^	−0.69 ± 0.18 ^b^	12.51 ± 0.28 ^a^
MI	5	89.26 ± 1.05 ^c^	−0.63 ± 0.09 ^bc^	12.50 ± 0.39 ^a^
MI	10	90.18 ± 0.25 ^b^	−0.55 ± 0.03 ^c^	12.22 ± 0.15 ^ab^
S		**	**	*
T		ns	ns	ns
S*T		ns	ns	ns

^a–c^ Values in the same column bearing different letters are different at *p* < 0.05. The values shown are mean ± SD. Abbreviations: MC, whole-milk Mozzarella cheese control; MI, reduced-fat Mozzarella cheese with inulin; ns, not significant; S, type of sample; T, time of storage; *, *p*-value < 0.05; **, *p*-value < 0.001.

## Data Availability

The original contributions presented in this study are included in the article. Further inquiries can be directed to the corresponding author.

## Abbreviations

The following abbreviations are used in this manuscript:

DP	Degree of polymerization
LEO	Laurel essential oil
MC	Whole-milk Mozzarella cheese
MI	Reduced-fat Mozzarella cheese with inulin
MUFAs	Monounsaturated fatty acids
PUFAs	Polyunsaturated fatty acids
SD	Standard deviation
SFAs	Saturated fatty acids
TCATA	Temporal Check-All-That-Apply
TPA	Texture profile analysis
